# Molecular Lesions of Insulator CTCF and Its Paralogue CTCFL (BORIS) in Cancer: An Analysis from Published Genomic Studies

**DOI:** 10.3390/ht7040030

**Published:** 2018-10-01

**Authors:** Ioannis A. Voutsadakis

**Affiliations:** 1 Algoma District Cancer Program, Sault Area Hospital, Sault Ste. Marie, ON P6B 0A8, Canada; ivoutsadakis@nosm.ca; 2 Section of Internal Medicine, Division of Clinical Sciences, Northern Ontario School of Medicine, Sudbury, ON P3E 2C6, Canada

**Keywords:** CTCF, CTCFL, BORIS, insulator, cancer, mutations, amplifications, topologically associating domains

## Abstract

CTCF (CCCTC-binding factor) is a transcription regulator with hundreds of binding sites in the human genome. It has a main function as an insulator protein, defining together with cohesins the boundaries of areas of the genome called topologically associating domains (TADs). TADs contain regulatory elements such as enhancers which function as regulators of the transcription of genes inside the boundaries of the TAD while they are restricted from regulating genes outside these boundaries. This paper will examine the most common genetic lesions of CTCF as well as its related protein CTCFL (CTCF-like also called BORIS) in cancer using publicly available data from published genomic studies. Cancer types where abnormalities in the two genes are more common will be examined for possible associations with underlying repair defects or other prevalent genetic lesions. The putative functional effects in CTCF and CTCFL lesions will also be explored.

## 1. Introduction

The three-dimensional organisation of DNA in interphase cell nuclei is important for the regulation of gene transcription [1]. DNA in human chromosomes is organised into higher-order domains called topologically associating domains (TADs) and these have subdomains called insulated neighbourhoods [2,3]. Protein CTCF (CCCTC-binding factor, also known as MRD21, Mental Retardation 21) defines borders of these domains in human genome.

The *CTCF* gene (Gene ID: 10664, Ensembl gene: ENSG00000102974) is located at human chromosome 16q22.1 region and has 13 exons spanning over 76,779 nucleotides according to GenBank. Its specific coordinates in the human genome assembly GRCh38 are chromosome 16: 67.562.407–67.639.185, forward strand. Alternative mRNA splicing produces different isoforms with 17 variants listed in Ensembl. Two promoters of the human gene are listed in the Eukaryotic Promoter database [4].

**Figure 1:**
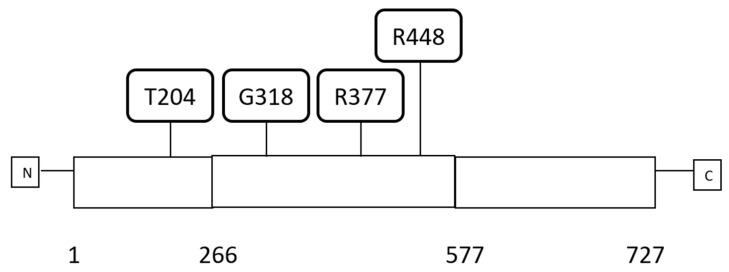
Schematic representation of CTCF protein with domain organisation and examples of the most frequent amino acid locations of recurrent mutations. Numbers below the protein schema represent margins of the protein domains. Domains are as follows: amino acids 1–266: amino-terminal domain, amino acids 267–577: Zinc finger domain, amino acids 578–727: carboxy-terminal domain. The amino acid locations of recurrent mutations are represented above the schematic. N: aminoterminal, C: carboxy-terminal.

CTCF protein (UniProtKB-P49711) has a length of 727 amino acids and is comprised of 11 C2H2-type zinc fingers (ZFs) occupying the central portion of the protein from amino acid 266 to amino acid 577 and flanked by an aminoterminal and a carboxyterminal domain that are both unstructured (Figure 1) [5]. CTCF ZFs are 23 to 24 amino acids long and accommodate the zinc atom through two cysteine and two histidine residues. CTCF is expressed ubiquitously in all human adult tissues. CTCF functions as a specific DNA sequence binding protein and has a role in gene transcription regulation both as a suppressor and an activator and regulates gene imprinting. CTCF plays also a major additional role in gene regulation by acting as a DNA topologically associating domains (TADs) insulator. In this capacity, it associates with the cohesin complex and another CTCF factor bound at a distance creating DNA loops. In many occasions, transcriptional activities mediated by enhancers are restricted inside these loops, insulating these enhancers from acting in transcription of genes outside the loop. TADs may also possess functional subdomains termed insulated neighbourhoods also defined by CTCF borders. Mutations affecting TADs or neighbourhoods borders in cancer may have profound effects in gene regulation by creating new influences by enhancers that were originally outside the TAD or outside the neighbourhood or conversely restricting enhancers from exerting normal regulations. There are thousands of potential CTCF binding sites in the human genome (possibly in the range of 11,000 to 14,000 and up to 60,000 in some studies), although it is unclear what percentage of these identify potential insulated neighbourhood borders [6,7,8]. The function of CTCF in gene imprinting is commonly illustrated with the example of the IGF2/H19 locus where, under the influence of CTCF differential binding, the IGF2 gene is only expressed by the paternal allele while H19 is exclusively expressed by the maternal allele. This involves also the insulator function of CTCF. Binding of CTCF to the locus is dependent on the DNA methylation status of CTCF binding sites in the neighbourhood of the two alleles which modifies the length of the insulated neighbourhood that includes the two genes and includes or excludes different enhancers from regulating each of the two genes [9].

CTCF paralogue, CTCFL (CCCTC-binding factor-Like, also known as BORIS, Brother of the Regulator of Imprinted Sites or CT27: Cancer/Testis antigen 27) is transcribed from a gene in human chromosome 20q13.31 (Gene ID: 140690, Ensembl gene: ENSG00000124092). *CTCFL* specific coordinates in the human genome assembly GRCh38 are chromosome 20: 57.495.966–57.525.652, reverse strand. It has 16 exons and 22 splice variants according to Ensembl.

The CTCFL protein (UniProtKB-Q8NI51) consists of 663 amino acids and, in contrast to the ubiquitous expression of its paralogue CTCF, is normally expressed only during spermatogenesis [10]. Similarly to CTCF, CTCFL possesses 11 C2H2-type zinc fingers that are highly homologous to those of CTCF, are 23 to 24 amino acids long, and occupy the middle portion of the protein from amino acid 257 to amino acid 568. The DNA binding sequence of CTCFL and CTCF is very similar but their capacity to interact with partner proteins is not conserved due to significant divergence in their aminoterminal and carboxyterminal domains. Most CTCFL binding sites are shared with CTCF but the reverse is not true as CTCF binds to ten times more sites [11]. The two paralogues may have differences in their ability to bind methylated sites with CTCFL being able to bind methylated sites while CTCF preferring unmethylated sites [12]. However, mere loss of methylation in a target site is not sufficient for CTCF binding in most occasions [13]. Normal CTCFL expression is restricted to spermatogonia and preleptotene spermatocytes. CTCFL expression becomes silenced in late spermatogenesis through promoter methylation and remains absent or very low in most adult tissues. Expression is reactivated in some cancer cases through promoter hypomethylation. Thus, CTCFL belongs to the category of so called cancer testis antigens, alternatively termed cancer-germline antigens [14]. Although details of the interaction are not known, CTCFL has been reported to be part of the CTCF interactome [15]. Whether the two proteins interact directly or indirectly in cells where they are co-expressed remains to be confirmed experimentally. The two paralogues have also been reported to cooperate in binding on tandem sites and thus it is possible that CTCF binding facilitates preferential CTCFL binding in an adjacent site [16].

This paper will investigate molecular lesions of CTCF and CTCFL in various cancers from published sources. Underlying molecular defects putatively associated with development of CTCF and CTCFL anomalies as well as prognostic implications of CTCF and CTCFL mRNA dosage will be explored.

## 2. Methods

Genomic studies of common cancers were interrogated in the cBioportal platform [17,18,19] for genetic lesions and mRNA dysregulation of the two paralogous genes of interest, *CTCF* and *CTCFL*. cBioportal contains several of the most extensive series of genomic studies performed by The Cancer Genome Atlas (TCGA) and other groups. The platform currently contains 226 genomic studies and allows for interrogation of each study for genetic lesions in any gene of interest. Genomic studies included in the cBioportal platform were examined for frequency and specific characteristics of cases with CTCF and CTCFL mutations and copy number alterations. Series with the higher absolute number of CTCF and CTCFL lesions were identified and examined in more detail to establish correlations with protein domain localisation of mutations and resulting total mutation burden. Studies selected for more detailed scrutiny included TCGA studies for endometrial, bladder, colon, and gastroesophageal carcinomas and the METABRIC breast cancer study [20,21,22,23,24]. These studies either contain the higher percentage of cases with *CTCF* and *CTCFL* lesions or, despite a lower percentage of lesions in these genes, the absolute numbers of defective cases were substantial in order to facilitate analysis. Studies of several other common types of cancers available in cBioportal were reviewed to determine frequency of *CTCF* and *CTCFL* defects [25,26,27,28,29,30,31,32].

Identified mutations were mapped in the different regions of each gene and assessed for their putative functional significance using the mutation assessor server, Computational Biology Center, Memorial Sloan Kettering Cancer Center, New York, NY, U.S.A. [33] which uses a multiple sequence alignment (msa) algorithm to assign a prediction score of functional significance to each mutation [34]. Additional investigations performed included identification of presence of MSI-related genes (MSH2, MSH6, PMS2, and MLH1) and polymerase *δ* and *ε* (POLD1 and POLE) defects in mutated samples and identification of the most commonly amplified region (amplicon) in samples with amplifications.

Survival of patients with high expression of CTCF and CTCFL mRNA versus those with low CTCF and CTCFL mRNA expression in examples of gastric, breast, and ovarian cancers was compared using the online tool Kaplan Meier Plotter [35,36].

Promoter methylation status was examined using published TCGA data and the online database for DNA methylation in cancer [37,38]. This database provides comparisons of methylation of promoter sequences of cancer samples of various cancers with corresponding sequences of respective normal tissues.

Categorical and continuous data were compared with the Fisher’s exact test and the *t*-test respectively. Correlations were explored with the Pearson correlation coefficient. All statistical comparisons were considered significant if *p* < 0.05.

## 3. Results

### 3.1. Molecular Lesions of CTCF in Cancer

**Figure 2:**
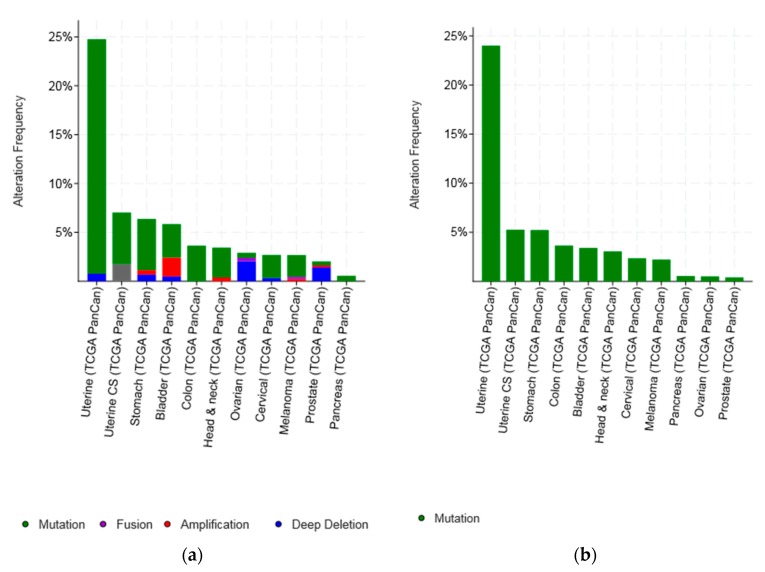
Percentage of CTCF mutations and amplifications combined (**a**) and mutations (**b**) in 12 common cancers. The total number of samples examined was 6043.

An overview of lesions of *CTCF* gene in various cancers studied by TCGA and in the METABRIC breast cancer study shows that the most common type of genetic lesions is mutations (1.97% of all samples examined), while amplifications and deep deletions were very rare (0.18% and 0.49%, respectively) (Figure 2a and Table 1). Cancers presenting with the higher percentage of lesions in *CTCF*: Uterine endometrial carcinomas (37.25% of total samples have *CTCF* lesions), ovarian serous carcinomas (16.5% of total samples with *CTCF* lesions), bladder carcinomas (13.61% of total samples with *CTCF* lesions), colorectal carcinomas (11.24% of total samples with *CTCF* lesions), and prostate cancers (10.79% of total samples with *CTCF* lesions) (Table 1). However, even mutations are rare and observed in less than 2% of samples in most types of cancer with the exception of endometrial, gastroesophageal, colorectal, bladder, and breast cancers (Figure 2b). Importantly, the type of cancer that stands out as having the highest mutation rate of *CTCF* is endometrial cancer, where *CTCF* is mutated in more than one fourth of tumours (27.45%). Uterine carcinosarcomas and gastric cancers display a mutation rate of *CTCF* of approximately 5% followed by colorectal, bladder, and breast cancers which display mutation rates of *CTCF* in 4.87%, 3.22%, and 2.21% of cases examined, respectively (Figure 2b).

Most mutations (59%) of *CTCF* gene in endometrial carcinomas in the uterine TCGA PanCancer study were located in the area encoding the 11 ZFs of the protein (23 of 39 samples with *CTCF* mutations in cases that had complete mutations, copy number alterations, and mRNA expression analysis data available, Table 2) [23]. The rest of the samples, except one that had a mutation in the C-terminal domain, had mutations in the N-terminal domain. Twenty-six of 39 *CTCF* gene mutated samples (66.7%) had one or more mutations in one of the four microsatellite instability (MSI)-associated genes (MSH2, MSH6, PMS2, and MLH1). An alternative cause of hypermutation in cancer is mutations in polymerases epsilon (POLE) and delta 1 (POLD1) [39]. Among the 39 *CTCF* mutated samples 23 samples had a concomitant mutation in one of these polymerases (Table 2). Overall 29 samples of the 39 *CTCF* mutated samples (74.4%) had mutations in MSI or the two polymerases. The mean number of total mutations in samples with mutations in MSI genes or the two polymerases was over 5000 while the 10 samples without mutations in these genes had a mean number of 301 mutations. These data suggest that *CTCF* mutations are commonly but not exclusively seen in MSI-associated endometrial carcinomas. A recurrent *CTCF* mutation in the N-terminal domain of *CTCF* in endometrial carcinomas observed in six samples was a frameshift mutation at codon T204 producing truncation of the protein after 18 or 26 amino acids. This recurrent mutation was not always associated with MSI or polymerase mutations. In only three of these cases there were concomitant MSI-associated gene mutations and one had polymerase mutations (Table 2) suggesting that even the exact same mutation may be associated or caused by various underlying molecular defects. Interestingly, no association was observed with APOBEC3 mRNA upregulation, which is also a cause of mutation induction in cancer [40,41]. This is a gene encoding for a DNA cytosine deaminase physiologically involved in the innate immune system-mediated protection against retroviruses and retrotransposons. Its function promotes mutagenesis through deamination of cytidines to uracils [40].

In colorectal cancer *CTCF* alterations are observed overall in 11.24% of cases and mutations are less frequent (4.87%). In the 13 samples with *CTCF* mutations in the Colorectal TCGA PanCancer study cohort [21] most mutations (12 of 13, 92.3%) were located in the ZFs or the N-terminal domain (Table 3). Most samples (10 of 13, 76.9%) had also mutations in one of the MSI-associated genes or polymerases POLE and POLD1 or both. Seven samples had over a thousand mutations per sample and all of these seven samples had mutations in one or both polymerases. Interestingly, many of the *CTCF* mutated samples, including two of the three samples without MSI-associated/polymerases mutations, had mutations in APOBEC genes (Table 3).

Urothelial carcinomas from the Bladder TCGA PanCancer study [22] was analysed in more detail as an example of cancer not usually associated with MSI (Table 4). The total number of samples with MSI-associated mutations in this study was 35 (8.6%). Among the 13 samples with *CTCF* mutations only three (23.1%) had mutations in MSI-associated genes (one of those with concomitant POLE mutation). Five of the 13 samples with *CTCF* mutations had mRNA upregulation of one of the APOBEC genes or of AID (Activation Induced Deaminase), a deaminase of the same family.

The putative functional significance of *CTCF* mutations in endometrial cancer and in breast cancer (as an example of a cancer not associated with MSI-associated mutations) were evaluated using mutation accessor and OncoKB. Among 91 different *CTCF* mutations found in endometrial carcinoma in TCGA, 45 (49.45%) are listed as likely oncogenic (the rest has unknown oncogenic potential and some of them may prove to be oncogenic as data accumulate). The METABRIC breast cancer study identified 44 different *CTCF* mutations (2.1% of samples) of which 20 mutations (45.5%) are considered likely oncogenic. The OncoKB database of mutations maintained by Memorial Sloan Kettering Cancer Center (oncokb.org) lists six point mutations of *CTCF* (H284N/Y/P, R339W, R377H, and P378L) and truncating mutations and deletions as oncogenic because of likely protein loss of function (or switch of function in the case of R339W).

**Figure 3:**
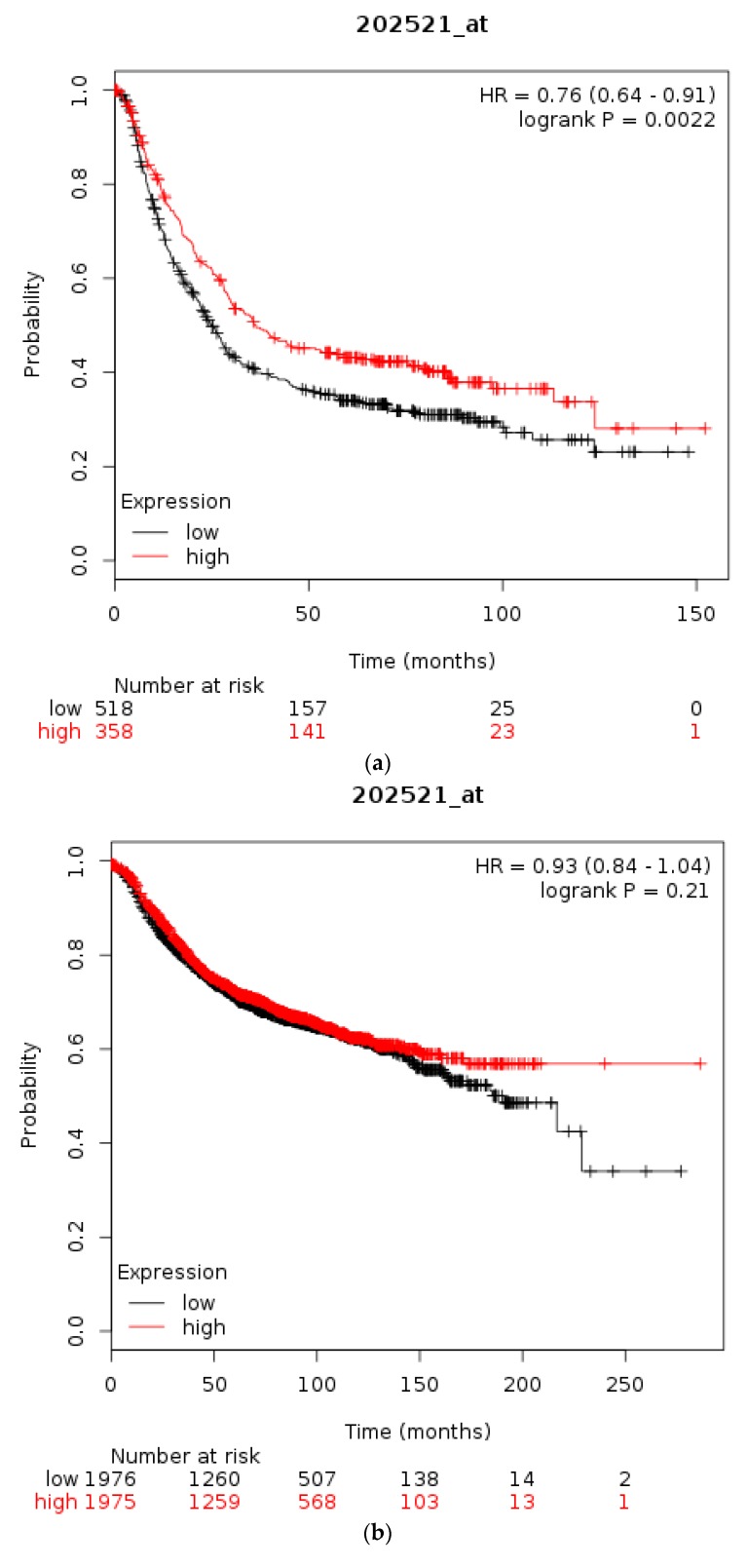

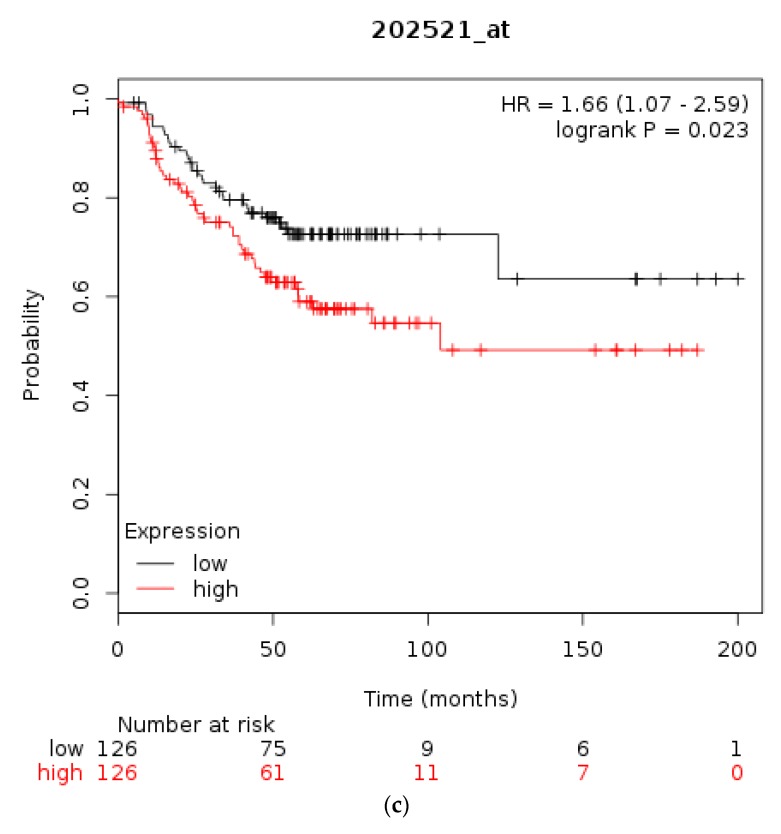
Overall survival of patients with high CTCF mRNA expression compared with patients with low CTCF mRNA expression. (**a**) Gastric cancer. (**b**) Breast cancer across all subtypes. (**c**) HER2+ Breast cancer. Low (black lines) and high (red lines) curves represent cases with mRNA levels below and above the mean of the groups.

The prognostic significance for survival of mRNA levels of CTCF in gastric, breast, and ovarian cancer were checked using Kaplan Meier plotter [31,35]. Gastric cancer patients with a high CTCF mRNA expression had an improved OS compared with counterparts with low CTCF mRNA expression (*HR* = 0.76, 95% *CI* = 0.64–0.91, *p* = 0.0022, Figure 3a). In breast cancer, overall across subtypes, CTCF mRNA levels are not associated with differences in OS (Figure 3b). However, in HER2-positive disease patients with a high CTCF mRNA expression displayed a worse OS compared with counterparts with low CTCF mRNA expression (*HR* = 1.66, 95% *CI* = 1.07–2.59, *p* = 0.023, Figure 3c). Similarly, in ER-positive patients high CTCF mRNA expression is associated with worse OS (not shown). In stage I and II ovarian cancer patients with a high CTCF mRNA expression also displayed a worse OS compared with counterparts with low CTCF mRNA expression (*HR* = 1.96, 95% *CI* = 1.02–3.78, *p* = 0.039, not shown). In contrast, in stage III and IV ovarian cancers there is no difference in OS between high and low CTCF mRNA levels groups (*p* = 0.13). These data suggest that CTCF levels are not directly associated with prognosis or may have different prognostic implications depending on the particular tumour.

### 3.2. Lesions of CTCFL in Cancer

**Figure 4:**
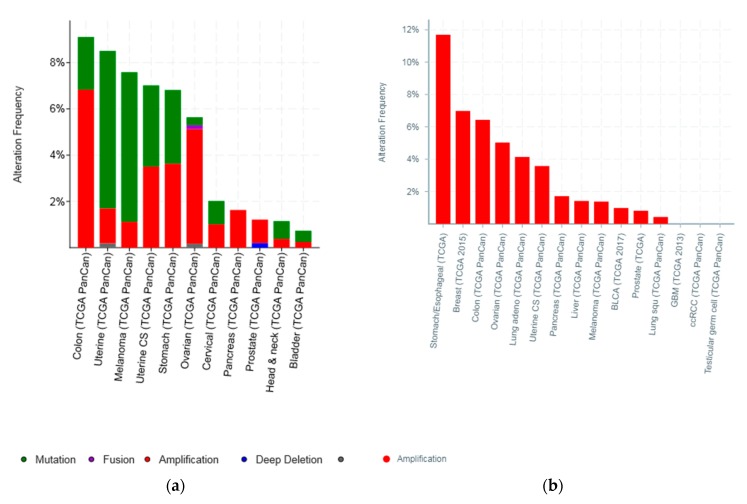
Percentage of CTCFL mutations and amplifications combined (**a**) and amplifications (**b**) in 12 common cancers. Please note that studies and types of cancer presented in parts (**a**) and (**b**) of the figure are only partially overlapping as the two parts include studies and cancer types with the higher prevalence of total CTCFL lesions and CTCFL amplifications which are different in each occasion.

Molecular lesions of *CTCFL* gene were observed in less than 10% of common tumours examined (Figure 4). However, several types of cancer had lesions in more than 6% of cases (Figure 4a). In addition to mutations, amplifications were common in *CTCFL*. Amplifications were the almost exclusive *CTCFL* lesion in breast cancer and constituted a significant percentage of *CTCFL* molecular lesions in ovarian, colon, and gastric carcinomas as well as uterine carcinosarcomas. On the other hand, melanomas and endometrial cancers showed mutations as the dominant type of lesion (Figure 4).

In the 10 samples with *CTCFL* mutations among samples that had complete mutations and copy number alterations information in the Colorectal PanCancer Atlas cohort [15] (Table 5), all had concomitant mutations in one of the four MSI associated genes or the *POLE* or *POLD1* genes, while only one of the 25 samples with *CTCFL* amplifications (used as a control) had such lesions in MSI-associated genes or *POLE*/*POLD1* genes (in *POLD1*) (Fisher’s two-tailed exact test *p* < 0.0001). In the respective endometrial carcinoma TCGA PanCancer Atlas study, which included 509 cases with complete mutations and copy number alterations information [23], among the 27 cases with *CTCFL* mutations 22 had mutations in one of the four MSI associated genes or the *POLE* or *POLD1* genes. In the nine samples with *CTCFL* amplifications two had mutations in those genes (one of the two had a concomitant *CTCFL* mutation) (Fisher’s two-tailed exact test *p* = 0.0025). In the cutaneous melanoma PanCancer Atlas study (provisional), 11 of 27 samples (40.7%) with *CTCFL* mutations had lesions in one of the four MSI associated genes or the *POLE* or *POLD1* genes. Thus, it appears that *CTCFL* mutations are often produced by underlying MSI or *POLE* or *POLD1* defects in both cancers commonly associated with these defects (colorectal and endometrial) and in other cancers less commonly associated with them (melanoma).

**Figure 5:**
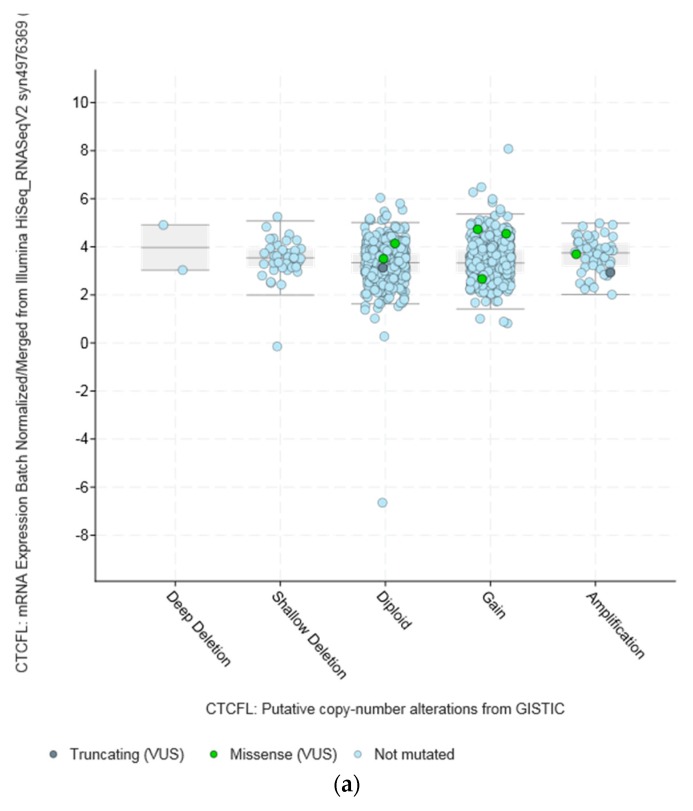

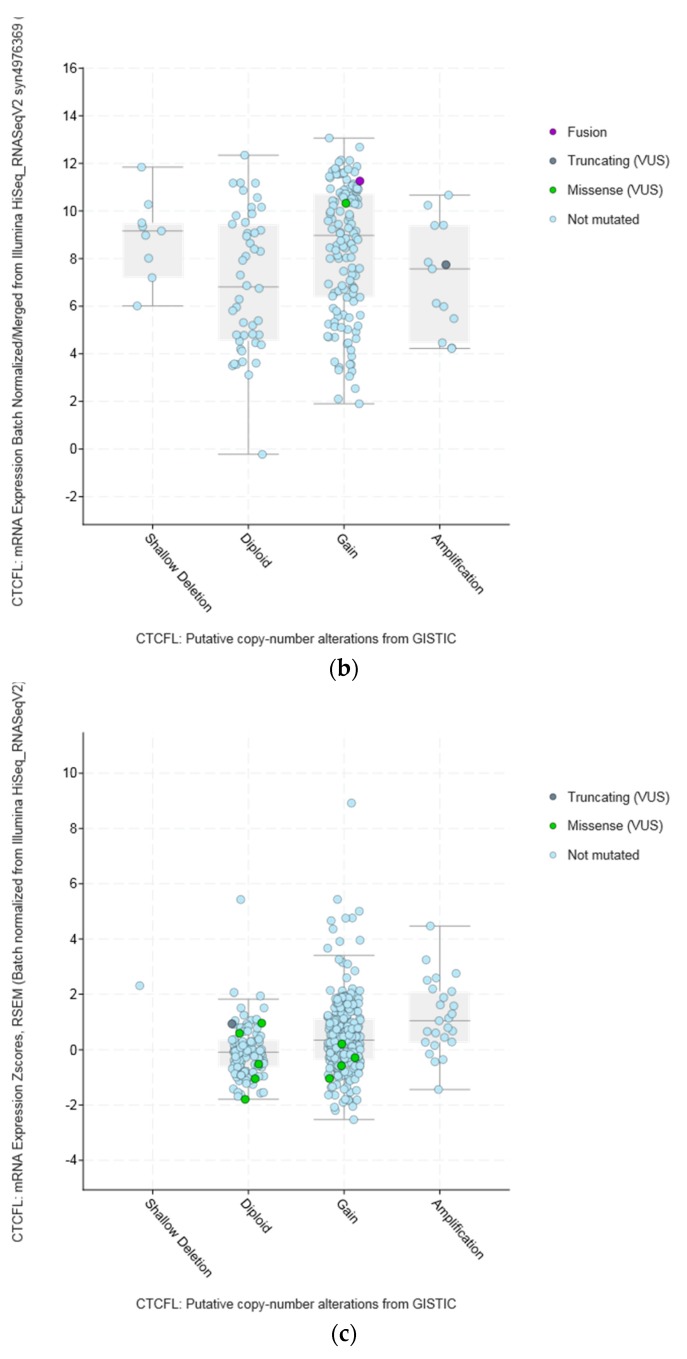

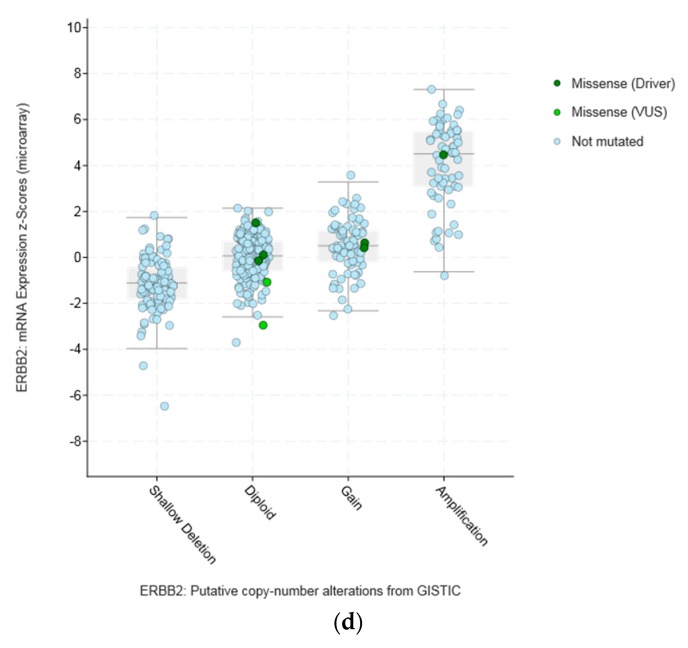
*CTCFL* gene amplification versus mRNA expression in various cancers. (**a**) Breast cancer. (**b**) Ovarian serous Cystadenocarcinoma. (**c**) Colon adenocarcinoma. (**d**) Gene amplification versus mRNA expression of ERBB2 in breast cancer. In (**a**,**b**) the mean mRNA levels in diploid and amplified cases are similar at 4 and 7, respectively. In (**c**,**d**) amplified cases present a higher mean mRNA level than diploid cases. GISTIC: Genomic Identification of Significant Targets in Cancer. The copy number analysis algorithm according to GISTIC defines a copy number below −2 as deep deletion (possible homozygous deletion), copy number between −2 and −1 as swallow deletion (possible heterozygous deletion), copy number between −1 and 1 as diploid, copy number between 1 and 2 as low-level gain, and copy number above 2 as amplification.

Amplifications of *CTCFL* do not always correlate with increased CTCFL mRNA expression. For example, mRNA expression in breast and ovarian cancers is not significantly increased in amplified cases (Figure 5a,b). In contrast in colon cancer *CTCFL* amplified samples display a higher CTCFL mRNA expression (Figure 5c). The mean normalised CTCFL mRNA expression of diploid colon cases was 19.46 (SD: 7.97) and the mean normalised CTCFL mRNA expression of amplified cases was 29.79 (*SD*: 10.84, *t* = 5.4, *p* = 0.001). However, there was no correlation of mean normalised CTCFL mRNA expression values with the Log2 copy number values in either breast or ovarian or colon cancers (Pearson correlation *p* 0.06, 0.07, and 0.26, respectively). As a comparison, amplifications of the *ERBB2* gene (encoding for the HER2 protein) in breast cancer result in increased mRNA expression compared with nonamplified tumours for *ERBB2* (Figure 5d). Moreover, CTCFL protein is rarely expressed in cancers, in contrast to CTCF that is ubiquitously expressed. The Human Protein Atlas [42] records an absence of CTCFL expression in all cancers examined, including cancers with comparatively high rate of CTCFL amplification such as gastroesophageal, breast, and colon (Figure 4b).

**Figure 6:**
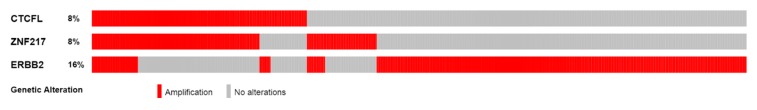
Amplifications of CTCFL, ZNF217, and ERBB2 in the METABRIC breast cancer study samples. Percentages represent the percentage of cases of each gene amplified in the study.

The amplicon of CTCFL at chromosome 20q13.31–32 contains, in addition to *CTCFL*, genes: *PCK1*, *PMEPA1* (also known as STAG1), *ZBP1*, *BMP7*, *MIR4325*, *MTRNR2L3*, *RAE1*, *RBM38*, *SPO11*, *MIR4532*, *C20ORF85*, and *ANKRD60*. All genes in the amplicon are amplified in a similar percentage of cases in different series, albeit in variable levels across cancers. In breast cancer, for example, all amplicon genes are amplified in approximately 6% to 7% in the TCGA PanCancer Atlas study and in 7% to 8% of cases in the METABRIC study (Figure S1) [19]. Thus, there is no clear indication of whether there is a driver gene among the amplicon genes that favours the amplification by promoting cancer cell fitness. Interestingly, some genes of the amplicon for which data are available in the human protein atlas, such as PCK1, PMEPA1, and RBM38, are expressed in the protein level at low to moderate levels in several cancers. Of additional interest is that the locus of 20q13 chromosomal region is commonly amplified in cancers but various subregions in this locus may be part of different amplicons. As an example, from the METABRIC study, Figure 6 shows that occurrence of *CTCFL* amplifications and amplifications of zinc finger transcription factor *ZNF217* (another zinc finger transcription factor located in a neighbouring locus at 20q13.2 and proposed to be an oncogene) are only partially overlapping in samples of breast cancer despite both being present in approximately 8% of cases. These amplifications are also partially overlapping with amplifications of *ERBB2* encoding for HER2 (Figure 6).

**Figure 7:**
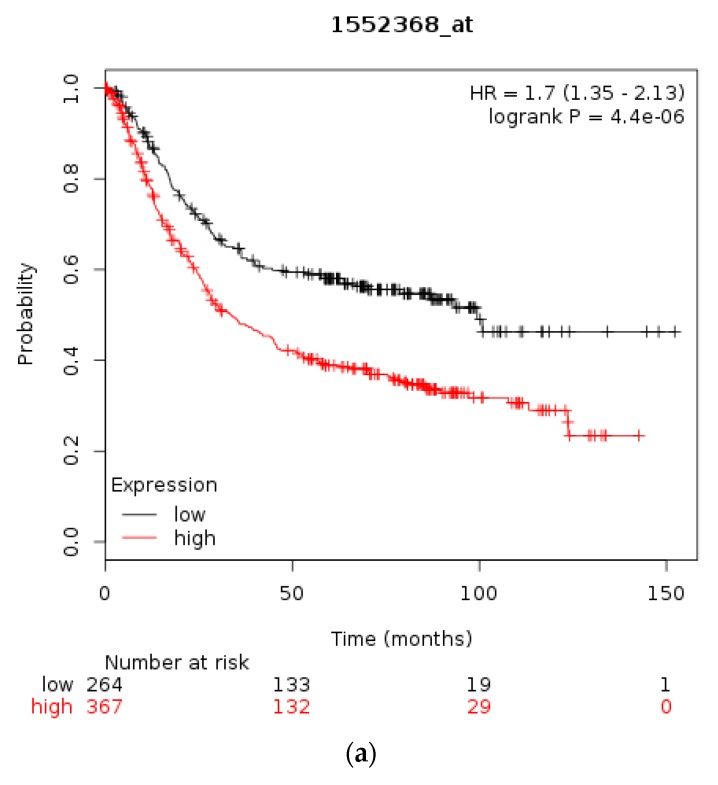

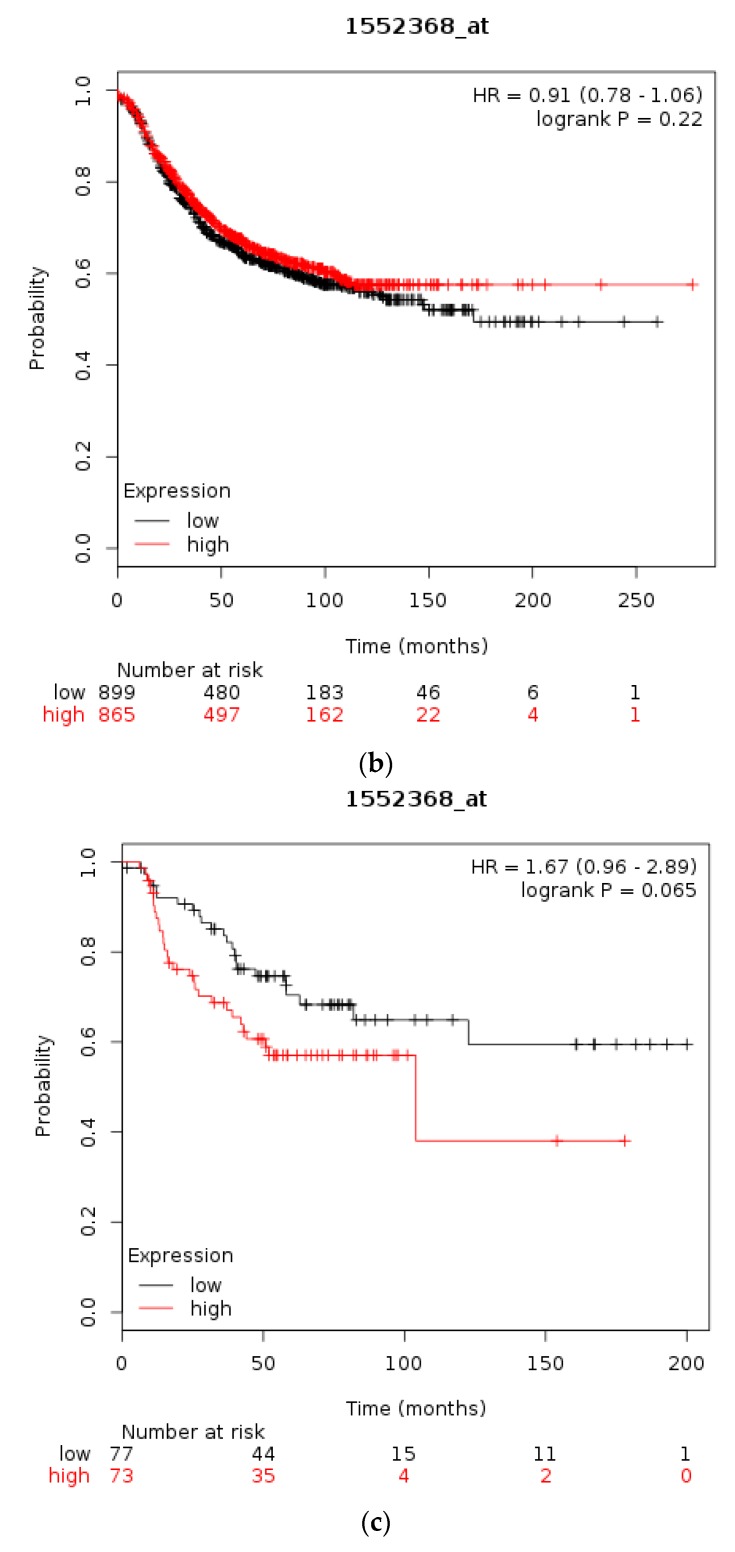
Overall survival of patients with high CTCFL mRNA expression compared with patients with low CTCFL mRNA expression. (**a**) Gastric cancer. (**b**) Breast cancer across all subtypes. (**c**) HER2+ Breast cancer. Low (black lines) and high (red lines) curves represent cases with mRNA levels below and above the mean of the groups.

Using the Kaplan Meier plotter [35] the prognostic significance of mRNA levels of CTCFL in gastric, breast, and ovarian cancer were interrogated, similarly to the respective levels of CTCF. In gastric cancer high CTCFL mRNA expression levels are associated with worse OS compared with gastric cancers having low CTCFL mRNA expression (*HR* = 1.7, 95% *CI* = 1.35–2.13, *p* = 0.000, Figure 7a). In breast cancer, independently of subtypes, survival of patients with high CTCFL mRNA levels is not different from OS of patients with low CTCFL mRNA levels (Figure 7b). HER2-positive breast cancers with a high CTCFL mRNA expression have a trend towards worse OS compared with counterparts with low CTCFL mRNA expression (*HR* = 1.67, 95% *CI* = 0.96–2.89, *p* = 0.065, Figure 7c). Patients with ER-positive breast cancer and high CTCFL mRNA expression have no difference in survival compared with low CTCFL mRNA expression counterparts (not shown). In stage I and II ovarian cancer patients with a high CTCFL mRNA expression displayed a worse OS compared with counterparts with low CTCF mRNA expression (*HR* = 2.66, 95% *CI* = 1.3–5.46, *p* = 0.0055, not shown). Similarly, stage III and IV ovarian cancers suffer worse survival when the CTCFL mRNA level of their tumours is high compared with patients with low levels (*HR* = 1.26, 95% *CI* = 1–1.59, *p* = 0.05). These data suggest that high CTCFL mRNA levels are commonly associated with adverse prognosis in various tumours, although there are exceptions such as ER-positive breast cancer.

**Figure 8:**
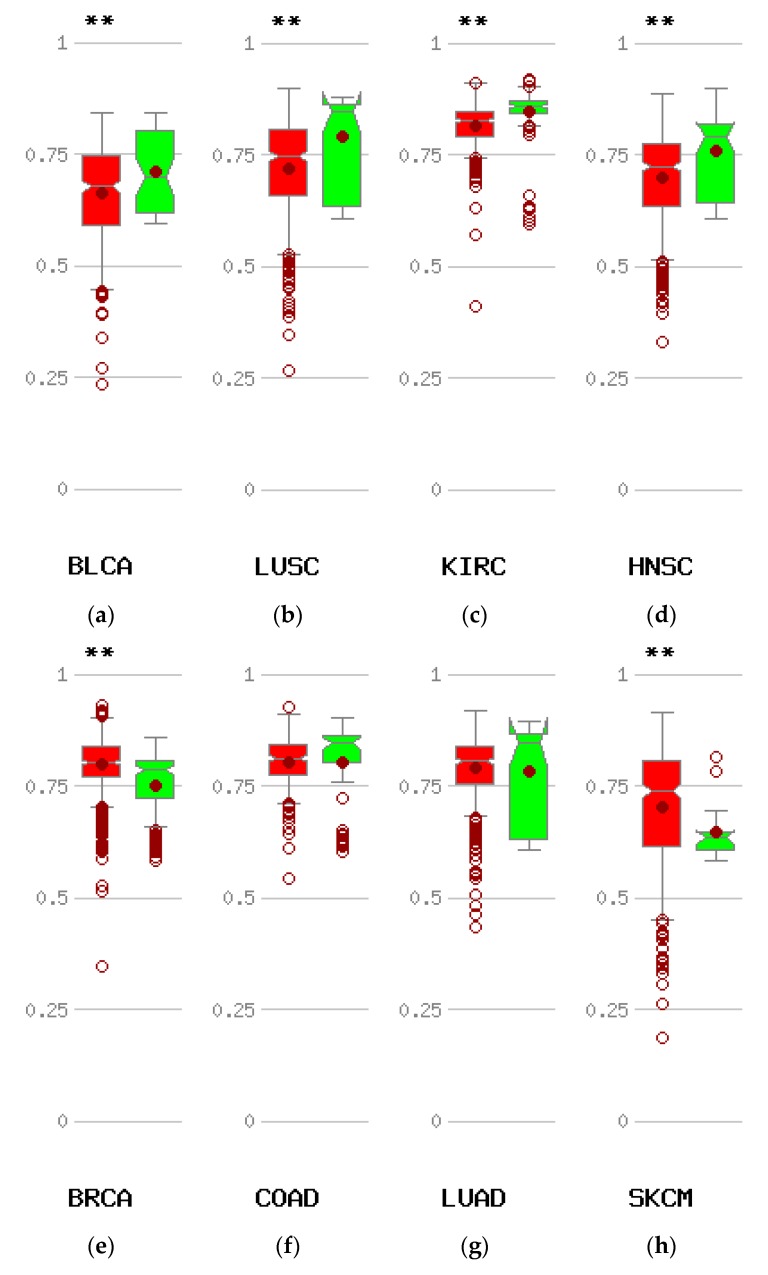
Methylation status of the CTCFL promoter in various cancers compared with corresponding normal tissues. Red symbols represent tumour samples and green normal samples. Numbers on the left of each comparison represent average beta values. ** *p* < 0.005. (**a**) Bladder carcinoma (BLCA). (**b**) Squamous carcinoma of the lung (LUSQ). (**c**) Clear cell Renal cell carcinoma (KIRC). (**d**) Head and neck cancer (HNSC). (**e**) Breast cancer (BRCA). (**f**) Colon cancer (COAD). (**g**) Adenocarcinoma of the lung (LUAD). (**h**) Melanoma (SKCM).

The most common mechanism causing re-expression of CTCFL in cancer is promoter hypomethylation. Data from the online platform MethHC comparing the methylation status of CTCFL promoters in various cancers with the status of these promoters in corresponding normal tissues disclose that compared with normal corresponding tissues, several common cancers such as carcinomas of the bladder, clear cell kidney, and squamous carcinomas of the lung, head, and neck display hypomethylation of *CTCFL* promoter (Figure 8a–d). However other common cancers such as colon and lung adenocarcinomas show no difference in their *CTCFL* promoter methylation status compared to normal colon and lung tissues (Figure 8f,g), while others, such as breast cancer and melanoma, even have promoter hypermethylation (Figure 8e,h).

## 4. Discussion

The CTCF transcription regulator is a main organizer of the human genome functioning as an insulator defining TADs borders. These act as physical barriers preventing function of remote enhancers from acting on genes outside the limits of the specific TAD. The insulating function of CTCF takes place through binding of the protein to specific DNA sequences that are ubiquitous throughout the human genome and recruitment of additional partner proteins interacting with the aminoterminal or the carboxyterminal domain of CTCF. Genetic lesions affecting either DNA binding of CTCF or interaction with partners could have severe implications for the function of CTCF as an insulator and lead to profound changes in the regulation of multiple genes through alterations in enhancer regulation, preventing enhancers from acting on normal target genes or creating new influences. These effects could be widespread throughout the genome. Upregulation of the expression of an oncogene or downregulation of a tumour suppressor under abnormal enhancer influences may promote cancer [43,44]. *CTCF* hemizygous mice displayed dysregulation of hundreds of cancer-related genes [45]. In this model of quantitative reduction of CTCF protein most affected were CTCF binding sites with weaker affinity for the protein. In another model of *CTCF* haploinsufficiency, using shRNA, decreased CTCF dose promotes cell survival and affects cell polarity, a hallmark of normal polarised epithelia [46]. *CTCF* mutations in endometrial carcinomas lead to nonsense-mediated decay of the transcripts or loss of function of the protein with missense mutations.

In this paper, *CTCF* DNA lesions were explored using published publicly available genomic studies and open platforms such as cBioportal available online. Several conclusions can be drawn from this investigation. First, *CTCF* lesions are rare across cancers, but mutations are much more common in certain cancers such as endometrial cancers than others. Second, underlying MSI-associated or polymerase mutations are common concomitant defects in *CTCF* mutant cases. This observation agrees with previous publications [47]. These authors have also observed that, similarly to the extensive published series included in the current report, MSI, although common, is not always present in *CTCF*-mutant cases. In some cases, *CTCF* mutations are associated with concomitant *POLE* or *POLD1* mutations. The two polymerases are responsible for the synthesis of the leading and lagging strand respectively during DNA replication and mutations in them lead to a hypermutator phenotype [48]. Mutations in *POLE* or *POLD1* lead to a polyposis syndrome called polymerase proofreading-associated polyposis (PPAP) [49]. Some cancers with lower MSI incidence may have other underlying defects that promote *CTCF* mutations, such as APOBEC deaminases abnormalities. Lastly, despite the fact that a significant proportion of *CTCF* mutations are considered oncogenic, the association of mRNA dose with tumour aggressiveness and prognosis is variable, suggesting that the protein is not a tumour suppressor in all contexts. This could be expected given the extensive role of CTCF in tertiary DNA organisation which leads to multiple gene dysregulations when defective. In addition, dosage of mRNA does not capture mutations which may have deleterious effects in protein function of an otherwise well-expressed protein. 

An additional function of CTCF consists of its involvement in double strand DNA repair [50,51]. Although the mechanism is not entirely clear, and whether involvement of PARP1, BRCA2, and RAD51 as repair partners required in this function is debated, CTCF appears to promote homologous recombination over nonhomologous end-joining (NHEJ) as the mechanism of double strand repair, thus favouring error-free DNA repair. As a result, point mutations in the protein or haplo-insufficiency due to nonsense mutations may have deleterious influence in double strand DNA repair which would be forced to proceed through the error-prone NHEJ mechanism. This could pose an additional burden for cancer cells with other repair defects such as MSI or promote errors creation even in cells that are microsatellite stable.

CTCF function may be more commonly affected through mutations in its ubiquitous DNA binding sites instead of mutations or other DNA alterations affecting the locus of the protein itself. Mutations in specific binding sites of CTCF may have effects in specific TADs but would be expected to have much less widespread influence on genome regulations than DNA lesions affecting the CTCF protein. On the other hand, such binding site mutations may have very specific oncogenic effects which may, for example, lead to expression of an oncogene under the influence of a new enhancer after TAD reshuffling. In agreement with this discussion, CTCF binding sites have been reported to be mutated at high frequency (25% and 19%, respectively) in gastric and colorectal cancers [52]. Moreover, in these cases, CTCF binding site mutations are commonly seen concomitantly with MSI.

CTCF paralogue CTCFL is normally not expressed in adult tissues, besides specific stages of spermatogenesis, due to promoter methylation of its gene, but is re-expressed in some cancer cases. In addition to epigenetic promoter hypomethylation that could promote CTCFL re-expression in cancer, genetic lesions may contribute to CTCFL de-repression. Abnormal re-expression of CTCFL in these cancer cases could have functional implications by interfering with binding of CTCF in a subset of its sites or by binding to methylated sites where CTCF may be less apt or cannot bind. Amplifications of *CTCFL* is the most common genetic lesion overall and could lead to overexpression of the protein. Data from the published studies presented in the current paper show that amplification is not always associated with mRNA upregulation (Figure 5) and thus the implications of such amplifications remain unclear. In addition, no other gene in the 20q13 amplicon appears to be more commonly amplified, a fact that would suggest cancer cell survival benefit leading to clonal dominance. Breast cancers with the common ERBB2 amplification defining the HER2-positive subset have only partially overlapping amplifications with *CTCFL* suggesting that the two amplicons may be created by different underlying mechanisms and not by a common mechanism affecting the two chromosomes, 17 and 20.

*CTCFL* promoter hypomethylation was evident in some types of cancers compared with corresponding normal tissues but not in other cancers (Figure 8). Whether there is a correlation of *CTCFL* promoter hypomethylation with protein expression in vivo in cancer patients remains untested. Squamous cell carcinomas of lung and head and neck which show *CTCFL* promoter hypomethylation (Figure 8b,d) have been observed to express CTCFL transcripts but no evidence exist for the corresponding protein expression [53]. Conversely, breast cancer displays hypermethylation in the *CTCFL* promoter (Figure 8e) and data suggest that CTCFL protein may not be expressed in human breast cancers, although this is controversial [54,55].

Some cancers present more commonly with CTCFL mutations rather than amplifications (Figure 4), and these could be of significance if the defective protein is expressed. In most occasions this is not the case and re-expression of CTCFL remains a rare occurrence in cancers. Thus, CTCFL mutations seem to be in many cases a marker of underlying genetic instability without pathologic implications of the specific mutation per se. Genetic instability such as MSI, *POLE*, and *POLD1* defects lead to an increased mutation burden which arises as a leading biomarker of response to immune blockade inhibitors, new drugs that have improved outcomes in several cancer types through immune stimulation. Besides responding better to these novel drugs, cancers with increased mutation burden tend also to have an improved prognosis. Given the significant effects that TAD border reshuffling may have for gene expression regulation, it would be of interest to further investigate the effect of CTCF and CTCFL lesions or their binding sites in TAD borders for refining the prognostic implications of mutation burden as a prognostic and immune checkpoint inhibitors predictive biomarker.

## Figures and Tables

**Table 1:** Summary of studies with CTCF lesions in various cancer studies. In the second column the number of samples with lesions is in the numerator and the total number of samples in the series is in the denominator. GBM: Glioblastoma multiforme, RCC: Renal cell carcinoma, HCC: Hepatocellular carcinoma, TGCTs: Testicular germ cell tumours, NA: Not available.

Type of Cancer (Reference)	All Lesions	Amplifications	Deep Deletions	mRNA Upregulation	mRNA Downregulation	Mutations	Multiple Lesions
Cell line encyclopedia [23]	57/877 (6.5%)	13 (1.48%)	6 (0.68%)	24 (2.74%)	14 (1.6%)	-	-
Uterine endometrial TCGA [23]	38/102 (37.25%)	-	1 (0.98%)	2 (1.96%)	3 (2.94%)	28 (27.45%)	4 (3.92%)
Ovarian serous TCGA [31]	33/200 (16.5%)	-	2 (1%)	3 (1.5%)	23 (11.5%)	1 (0.5%)	4 (2%), two fusions
Bladder TCGA [20]	55/404 (13.61%)	3 (0.74%)	1 (0.25%)	25 (6.19%)	8 (1.98%)	13 (3.22%)	5 (1.24%)
Colorectal TCGA [21]	30/267 (11.24%)	-	-	15 (5.62%)	2 (0.75%)	13 (4.87%)	-
Prostate TCGA [32]	53/491 (10.79%)	-	14 (2.85%)	12 (2.44%)	22 (4.48%)	3 (0.61%)	2 (0.41%)
Melanoma TCGA PanCancer (Provis.)	36/363 (9.92%)	-	-	14 (3.86)	11 (3.03%)	7 (1.93%)	4 (1.1%)
Lung adenocarcinoma TCGA [28]	40/503 (7.95%)	-	1 (0.2%)	21 (4.17%)	12 (2.39%)	5 (0.99%)	1 (0.2%)
Lung squamous TCGA [29]	34/466 (7.3)	-	-	19 (4.08%)	9 (1.93%)	4 (0.86%)	2 (0.42%), one fusion
Pancreatic TCGA PanCancer (Provis.)	12/168 (7.14%)	-	-	6 (3.57%)	5 (2.98%)	1 (0.6%)	-
TGCTs TCGA PanCancer (Provis.)	10/144 (6.94%)	-	-	6 (4.17%)	3 (2.08%)	-	1 (0.69%)
HCC TCGA PanCancer (Provis.)	20/345 (5.8%)	1 (0.29%)	-	6 (1.74%)	9 (2.61%)	4 (1.16%)	-
RCC TCGA [30]	20/352 (5.68%)	-	-	6 (1.7%)	13 (3.69%)	1 (0.28%)	-
Uterine serous TCGA [23]	3/53 (5.66%)	-	-	2 (3.77%)	1 (1.89%)	-	-
GBM TCGA [27]	5/142 (3.52%)	-	-	3 (2.11%)	2 (1.41%)	-	-
Breast TCGA [26]	26/816 (3.19%)	3 (0.37%)	5 (0.61%)	-	-	18 (2.21%)	-
Gastric adenocarcinoma TCGA [22]	4/188 (2.13%)	1 (0.53%)	1 (0.53%)	NA	NA	2 (1.06%)	-
Oesophageal adenocarcinoma TCGA [22]	1/77 (1.3%)	1 (1.3%)	-	NA	NA	-	-
All (Not cell lines)	420/5081 (8.27%)	9/5081 (0.18%)	25/5081 (0.49%)	140/4816 (2.9%)	123/4816 (2.55%)	100/5081 (1.97%)	22/5081 (0.43%)

**Table 2:** Endometrial cancer *CTCF* mutations in the TCGA PanCa study. All *CTCF* mutated samples were diploid. ZF: Zinc finger, N: Aminoterminal, C: Carboxyterminal, FS: Frameshift. No: No mutation, *: Stop codon.

Number of Sample	Protein Change	Domain	Mutation Type	Allele Frequency	Number of Mutations in Sample	Mutations in MSI-Associated Genes	Mutations in POLE/POLD1 Genes
1	G48Vfs*14	N	FS del	0.37	774	No	No
2	G48Vfs*14	N	FS del	0.28	716	MSH6	No
3	A88V	N	Missense	0.17	5737	MSH2, MSH6, MLH1, PMS2	POLE, POLD1
4	G173 *	N	Nonsense	0.25	7390	MSH2, MSH6, PMS2	POLE
5	Q180 *	N	Nonsense	0.4	594	MSH6	No
6	E182Gfs*9	N	FS ins	0.33	9662	MSH2, PMS2	POLE
7	T204Qfs*18	N	FS del	0.19	597	MLH1	No
8	T204Nfs*26	N	FS ins	0.26	538	MSH6, PMS2	No
9	T204Nfs*26	N	FS ins	0.35	323	No	No
10	T204Nfs*26	N	FS ins	0.5	864	PMS2	POLE
11	T204Nfs*26	N	FS ins	0.31	451	No	No
12	T204Nfs*26	N	FS ins	0.25	56	No	No
13	R213C	N	Missense	0.33	12218	MSH6, MLH1, PMS2	POLE, POLD1
14	D222Mfs*28	N	FS del	0.32	218	No	No
15	G261C	N	Missense	0.29	451	No	No
16	Q267P	ZF	Missense	0.32	13840	MSH2, PMS2, PMS1	POLE, POLD1
17	H312N	ZF	Missense	0.39	3190	MSH6, PMS2	POLE
18	T317Rfs*91	ZF	FS del	0.5	669	PMS2	No
19	G318Qfs*16	ZF	FS ins	0.22	562	No	No
20	C324 *	ZF	Nonsense	0.32	611	No	POLE
21	R341H	ZF	Missense	0.45	9662	MSH2, PMS2	POLE
22	R342C	ZF	Missense	0.4	9440	MSH2, MSH6, PMS1	POLE
23	Y343C	ZF	Missense	0.34	7644	MSH6, MLH1	POLE
24	H369R	ZF	Missense	0.17	1326	No	POLE
25	R377C	ZF	Missense	0.34	1307	MSH6	POLE, POLD1
26	P378L	ZF	Missense	0.2	13840	MSH2, PMS2	POLE, POLD1
27	L394del	ZF	IF del	0.09	716	MSH6	No
28	G420C	ZF	Missense	0.26	1307	MSH6	POLE, POLD1
29	E432Gfs*10	ZF	FS del	0.38	41	No	No
30	A447Vfs*61	ZF	FS del	0.45	4346	PMS1, MLH1	POLE, POLD1
31	R448 *	ZF	Nonsense	0.46	81	No	No
32	K449T	ZF	Missense	0.34	8511	MSH2, MLH1, PMS1	POLE
33	S450Kfs*2	ZF	FS ins	0.26	4096	MSH2, MSH6	POLE
34	X453_splice	ZF	Splice	0.28	58	No	No
35	R457 *	ZF	Nonsense	0.37	10061	MSH6, MSH2, PMS2	POLE, POLD1
36	Q499 *	ZF	Nonsense	0.35	3925	MSH6	POLE
37	R533H	ZF	Missense	0.47	611	No	POLE
38	R566H	ZF	Missense	0.2	884	MSH2, MSH6	POLD1
39	E631 *	C	Nonsense	0.41	3387	MSH6, MLH1	POLE

Notes: ZF: Zinc finger, N: Aminoterminal, C: Carboxyterminal, FS: Frameshift. No: No mutation, *: Stop codon.

**Table 3:** Colorectal cancer CTCF mutations. All samples were diploid except one which had CTCF DNA gain.

Number	Protein Change	Domain	Mutation Type	Allele Frequency	Number of Mutations in Sample	Mutations MSI	POLE/POLD1 Mutations	APOBEC Mutations
1	R29W	N	Missense	0.44	170	No	No	No
2	E182Gfs*9	N	FS ins	0.29	1858	No	POLD1	APOBEC2
3	D194Rfs*36	N	FS ins	0.25	814	No	No	APOBEC4
4	T204Qfs*18	N	FS del	0.1	530	MLH1, PMS1	No	APOBEC4
5	T204Nfs*26, D194_A201delinsTQTIS	N	FS ins, Missense	0.11, 0.17	1917	PMS1	POLE	APOBEC1
6	K260 *	N	Nonsense	0.25	181	No	No	APOBEC3B
7	R278C	ZF	Missense	0.24	1002	MSH2	POLE, POLD1	APOBEC3C
8	R368C	ZF	Missense	0.58	171	No	No	No
9	R377C	ZF	Missense	0.41	2139	MSH6, PMS2	POLE, POLD1	APOBEC4, APOBEC3C
10	E464K	ZF	Missense	0.28	1107	No	POLD1	APOBEC3A
11	G519R	ZF	Missense	0.18	662	No	POLD1	No
12	A524T	ZF	Missense	0.29	4195	MSH6	POLE, POLD1	APOBEC3G
13	E691Sfs*30	C	FS del	0.33	1057	No	POLD1	APOBEC3C

Notes: ZF: Zinc finger, N: Aminoterminal, C: Carboxyterminal, No: No mutation, *: Stop codon.

**Table 4:** CTCF mutations in urothelial cancer. Most mutations were in Zinc Fingers (ZF) domain of the protein. Seven samples were diploid and three each had gains and shallow deletions.

Number	Protein Change	Domain	Mutation Type	Number of Mutations in Sample	Mutations in MSI	Mutations in POLE/POLD1
1	E104 *, E145Q, K264N	N	Nonsense	580	No	No
2	Q117 *	N	Nonsense	58	No	No
3	D290N	ZF	Missense	291	No	No
4	H322Y	ZF	Missense	588	No	No
5	T346N	ZF	Missense	881	No	No
6	F351L	ZF	Missense	508	No	No
7	S354F	ZF	Missense	182	No	No
8	S354Y	ZF	Missense	124	No	No
9	G375A	ZF	Missense	857	MSH2	No
10	E376 *	ZF	Nonsense	133	No	No
11	S388N	ZF	Missense	283	No	No
12	E631 *	C	Nonsense	3545	MSH2, MLH1	POLE
13	E687 *	C	Nonsense	766	MSH6	No

Notes: N: Aminoterminal, C: Carboxyterminal, *: Stop codon.

**Table 5:** Summary of CTCFL lesions in various cancer studies. GBM: Glioblastoma multiforme, RCC: Renal cell carcinoma, HCC: Hepatocellular carcinoma, TGCTs: Testicular germ cell tumours, NA: Not available.

Type of Cancer (Reference)	All Lesions	Amplifications	Deep Deletions	mRNA Upregulation	mRNA Downregulation	Mutations	Multiple Lesions
Cell line encyclopedia [25]	100/877 (11.4%)	79 (9.01%)	-	19 (2.17%)	-	-	2 (0.23%)
Uterine serous TCGA [23]	17/53 (32.08%)	4 (7.55%)	-	13 (24.53%)	-	-	-
Ovarian serous TCGA [31]	37/200 (18.5%)	12 (6%)	-	22 (11%)	-	1 (0.5%)	2 (1%) 1 fusion
Gastric adenocarcinoma TCGA [22]	26/188 (13.83%)	24 (12.77%)	-	NA	NA	2 (1.06%)	-
Melanoma TCGA PanCancer (Provisional)	49/363 (13.5%)	4 (1.1%)	-	17 (4.68%)	-	27 (7.44%)	1 (0.28%)
Colorectal TCGA [21]	33/267 (12.36%)	9 (3.37%)	-	13 (4.87%)	-	5 (1.87%)	6 (2.25%)
Uterine endometrial [23]	12/102 (11.76%)	-	-	3 (2.94%)	-	7 (6.86%)	2 (1.96%)
Lung adenocarcinoma TCGA [28]	54/503 (10.74%)	19 (3.78%)	1 (0.2%)	13 (2.58%)	-	19 (3.78%)	2 (0.4%)
Esophageal adenocarcinoma TCGA [22]	7/77 (9.09%)	7/77 (9.09%)	-	NA	NA	-	-
Breast TCGA [26]	62/816 (7.6%)	56 (6.86%)	2 (0.25%)	-	-	3 (0.37%)	-
Lung squamous TCGA [29]	33/466 (7.08%)	1 (0.21%)	1 (0.2%)	22 (4.72%)	-	8 (1.72%)	1 (0.2%)
Prostate TCGA [32]	32/491 (6.52%)	4 (0.81%)	1 (0.2%)	13 (2.65%)	12 (2.44%)	2 (0.41%)	-
TGCTs TCGA PanCancer [Provisional]	9/144 (6.25%)	-	-	9 (6.25%)	-	-	-
Pancreatic TCGA PanCancer (Provisional)	10/168 (5.95%)	1 (0.6%)	-	6 (3.57%)	1 (0.6%)	-	2 (1.19%)
HCC TCGA PanCancer (Provisional)	19/345 (5.51%)	5 (1.45%)	-	12 (3.48%)	-	2 (0.58%)	-
GBM TCGA [27]	6/142 (4.23%)	-	-	6/142 (4.23%)	-	-	-
RCC TCGA [30]	10/352 (2.84%)	-	-	8 (2.27%)	1 (0.28%)	1 (0.28%)	-
Bladder TCGA [20]	10/404 (2.48%)	4 (0.99%)	-	3 (0.74%)	-	3 (0.74%)	-
All (Not lines)	426/5081 (8.38%)	150/5081 (2.95%)	5/5081 (0.1%)	160/4816 (3.3%)	14/4816 (0.29%)	80/5081 (1.57%)	16/5081 (0.31%)

## Supplementary Materials

The following are available online at http://www.mdpi.com/2571-5135/7/4/30/s1, Figure S1: The CTCFL amplicon in the METABRIC breast cancer study.
