# Supplementary material for: Molecular Lesions of Insulator CTCF and Its Paralogue CTCFL (BORIS) in Cancer: An Analysis from Published Genomic Studies
Source: High Throughput. 2018 Oct 1;7(4):30. doi: 10.3390/ht7040030 (PMC6306835; doi:10.3390/ht7040030)
Supplement: Supplementary file 1 [file high-throughput-07-00030-s001.pdf]

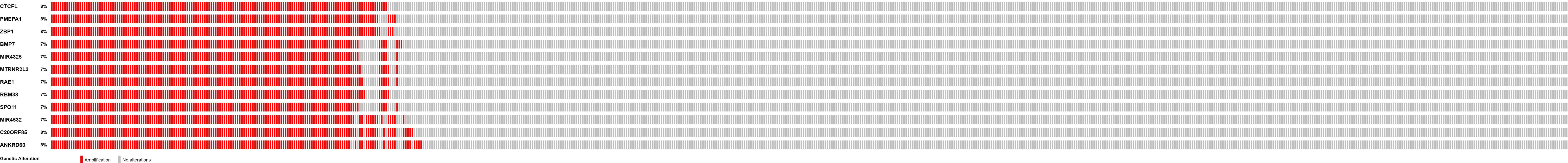
CTCFL 8%
PMEPA1 8%
ZBP1 8%
BMP7 7%
MIR4325 7%
MTRNR2L3 7%
RAE1 7%
RBM38 7%
SPO11 7%
MIR4532 7%
C20ORF85 8%
ANKRD60 8%
Genetic Alteration
Amplification
No alterations
